# Characterization of subgingival plaque microbiota in patients with severe periodontitis using full-length 16S rRNA gene sequencing

**DOI:** 10.1038/s41598-025-30064-8

**Published:** 2025-12-02

**Authors:** Jiale Ma, Shinya Kageyama, Mikari Asakawa, Michiko Furuta, Yoshihisa Yamashita, Toru Takeshita

**Affiliations:** Section of Preventive and Public Health Dentistry, Division of Oral Health, Growth and Development, Faculty of Dental Science, Kyushu University, Fukuoka, Japan

**Keywords:** Periodontitis, Amplicon sequence variant, Tongue microbiota, Plaque microbiota, 16S rRNA gene, PacBio long-read sequencing, Diseases, Microbiology

## Abstract

Periodontitis is a widespread global health issue with significant systemic health implications. Subgingival plaque microbiota is considered its driving factor. Thus, accurate profiling of the microbiota is essential for understanding the etiology of periodontitis. We investigated the subgingival plaque, supragingival plaque, and tongue microbiota in 34 patients with severe periodontitis using PacBio long-read sequencing of the full-length 16S ribosomal RNA gene and amplicon sequence variant (ASV) analysis. The alpha and beta diversities of subgingival and supragingival plaque microbiota were distinct from those of tongue microbiota. Despite their distinct bacterial compositions, the tongue and plaque microbiota shared an average of 20 ASVs per participant, accounting for approximately 13% and 38% of the tongue and plaque microbiota, respectively. Comparative microbiota analysis across these oral niches identified 17 species that were specifically enriched in the subgingival plaque. They accounted for 18.9 ± 20.2% (mean ± SD) of the subgingival plaque microbiota and predominantly consisted of *Porphyromonas gingivalis*, *Parvimonas micra*, and *Mogibacterium timidum*. Altogether, we demonstrate a high-resolution profile of the subgingival microbiota, thereby providing information to improve our understanding of the complex etiology of periodontitis.

**Supplementary Information:**

The online version contains supplementary material available at 10.1038/s41598-025-30064-8.

## Introduction

 Periodontal disease is a global health issue affecting a substantial proportion of the population^1^. Periodontitis is a chronic inflammatory disease that primarily damages the supporting tissues surrounding the teeth, including the gingiva, periodontal ligament, and alveolar bone, and can cause tooth mobility and eventual tooth loss^2^. Furthermore, periodontitis is associated with systemic diseases, such as cardiovascular diseases, aspiration pneumonia, and cancer, emphasizing its potential impact on overall health^3^.

Subgingival microbiota, a community of microorganisms residing in the gingival sulcus or periodontal pockets, has been implicated in the onset and progression of periodontitis by inducing harmful inflammatory responses in the host^4,5^. Therefore, numerous previous studies have attempted to profile the subgingival microbiota to elucidate the etiology of periodontal disease^6–10^. The advent of the PacBio sequencing system and full-length 16S ribosomal RNA (rRNA) sequencing offer new opportunities for studying microbial communities with high accuracy and resolution^11,12^. Although full-length PacBio sequencing has been applied to periodontal and peri-implant samples in a few studies, its potential in oral microbiome research, particularly for analyzing subgingival plaque microbiota, remains underexplored^13–17^. Accurate identification of subgingival bacterial species could provide key information about the pathogens associated with periodontitis and deepen our understanding of the etiology of periodontal disease.

The difficulty in profiling the subgingival microbiota is partly due to the proximal presence of supragingival plaque and the difficulty in discriminating bacterial species from the subgingival specimen. We collected supragingival plaque (SUPP), subgingival plaque (SUBP), and tongue coating (TC) samples from 34 Japanese adults diagnosed with severe periodontitis who visited dental clinics. A comparison of the subgingival microbiota with other niche microbiota, particularly those of supragingival microbiota, enabled the identification of bacterial species specific to subgingival plaque. The relationship between microbiota in different oral niches has not yet been determined using full-length PacBio sequencing. We accurately determined the bacterial composition of SUPP, SUBP, and TC samples using full-length 16S rRNA gene sequencing and an amplicon sequence variant (ASV) approach, and characterized the subgingival plaque microbiota by comparing them.

## Results

### Participants’ characteristics and full-length 16S rRNA gene sequences

We examined 34 participants (14 men and 20 women, aged 29–77 years) with ≥ 20% of probing sites with probing depth (PD) ≥ 4 mm, who visited eight dental clinics in Japan. Their detailed characteristics are summarized in Table 1. The mean number of present teeth was 25.6 ± 3.8 teeth (mean ± SD). Notably, all participants exhibited a high percentage of periodontal sites with PD ≥ 4 mm (53.9 ± 19.4%), and the mean percentage of bleeding on probing (BOP) sites was 60.4 ± 25.1%. A total of 102 samples were analyzed using PacBio long-read sequencing with full-length 16S rRNA gene amplicon analysis to determine the bacterial composition. Denoising with DADA2^18^ resulted in 792,354 denoised reads (4,893 ± 2,803 reads per SUBP sample, 5,172 ± 3,918 reads per SUPP sample, and 13,239 ± 5,490 reads per TC sample) and 7,835 ASVs. Of all ASVs, 7,412 ASVs (97.7% of all reads) with ≥ 98.5% identity with the reference sequences in eHOMD were clustered into 446 species-level taxa.

**Table 1:** Characteristics of the study participants.

	Participants (*n* = 34)
Age	54.1 ± 12.4
Male	14 (41.2)
Number of present teeth	25.6 ± 3.8
Number of PD4 sites	83.7 ± 33.7
Percentage of PD4 sites	53.9 ± 19.4
Number of BOP sites	94.1 ± 42.6
Percentage of BOP sites	60.4 ± 25.1

Data are presented as mean ± standard deviation and n (%). PD4, probing depth ≥ 4 mm; BOP, bleeding on probing.

### Microbial differences and the link between tongue and plaque microbiota

**Fig. 1:**
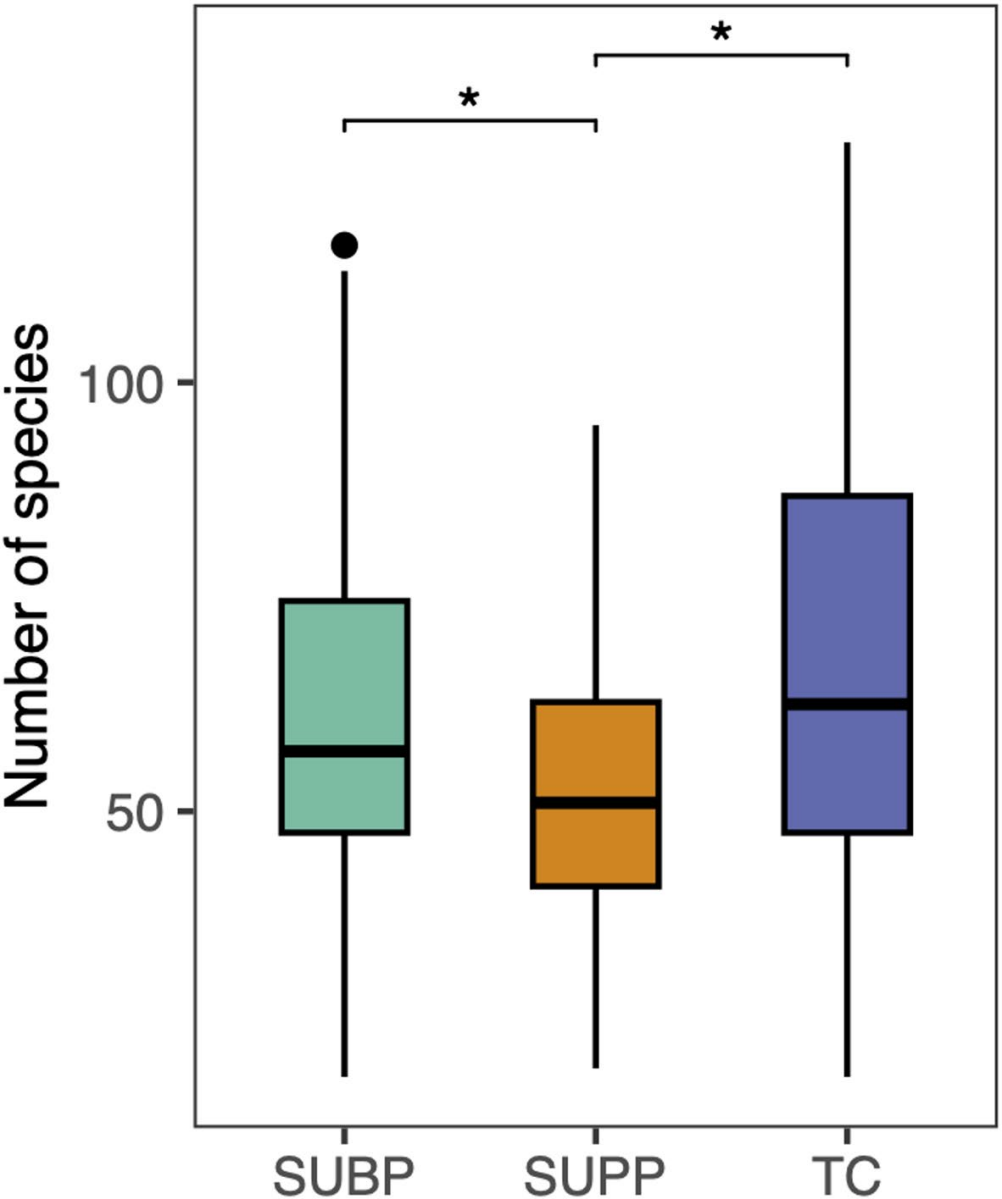
Comparison of alpha diversity across subgingival plaque (SUBP), supragingival plaque (SUPP), and tongue coating (TC) microbiota. Alpha diversity was assessed using the number of species in samples collected from 34 patients with severe periodontitis. Statistical differences between niches were analyzed using the Wilcoxon signed-rank tests with Benjamini–Hochberg false discovery rate (FDR) adjustment. *P < 0.05.

**Fig. 2:**
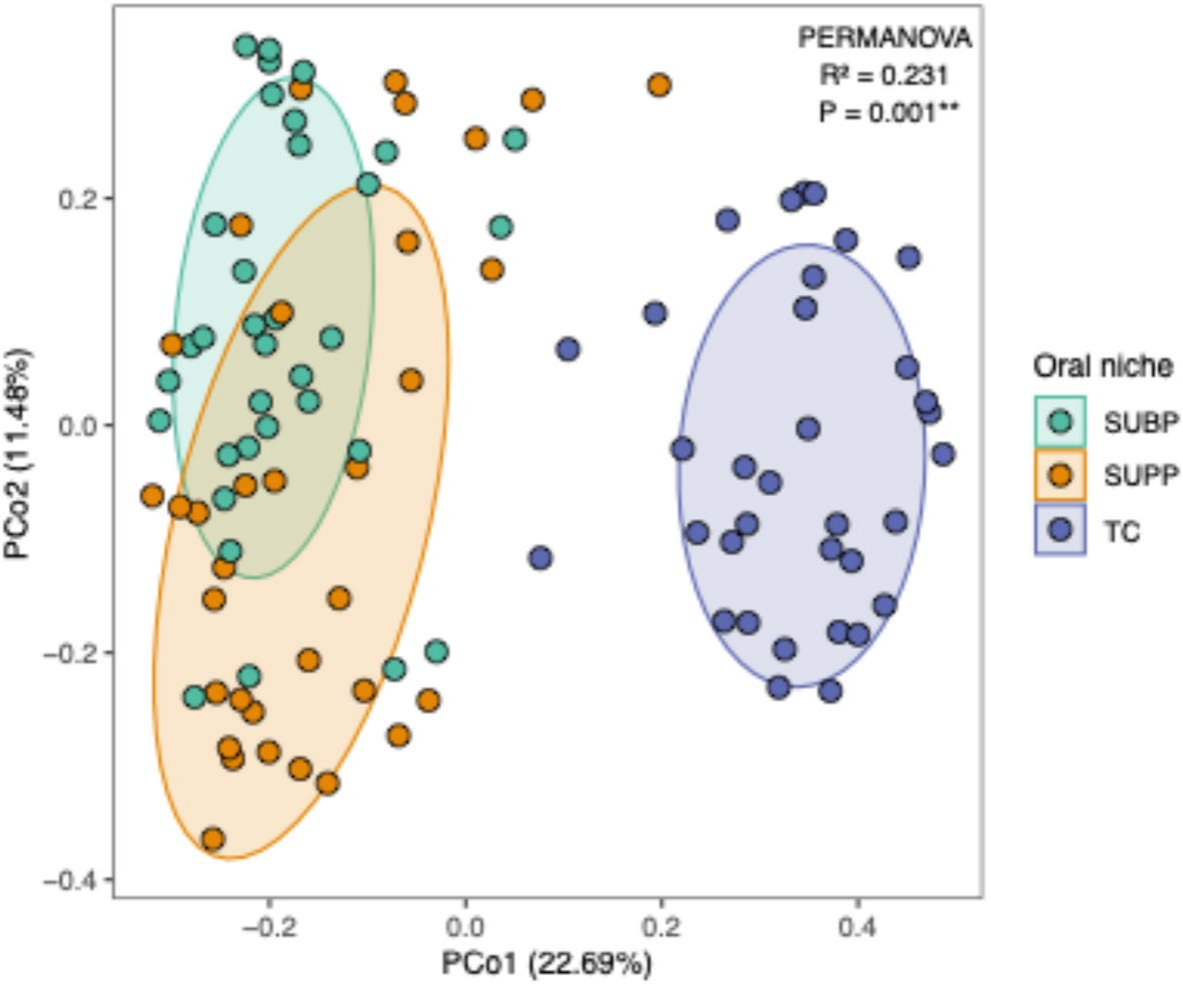
Principal coordinate analysis (PCoA) plot based on the log-transformed Bray–Curtis distance at the species level. Samples from subgingival plaque (SUBP), supragingival plaque (SUPP), and tongue coating (TC) are depicted in different colors (*n* = 34). Ellipses represent 67% confidence intervals for each niche. **P < 0.01.

**Fig. 3:**
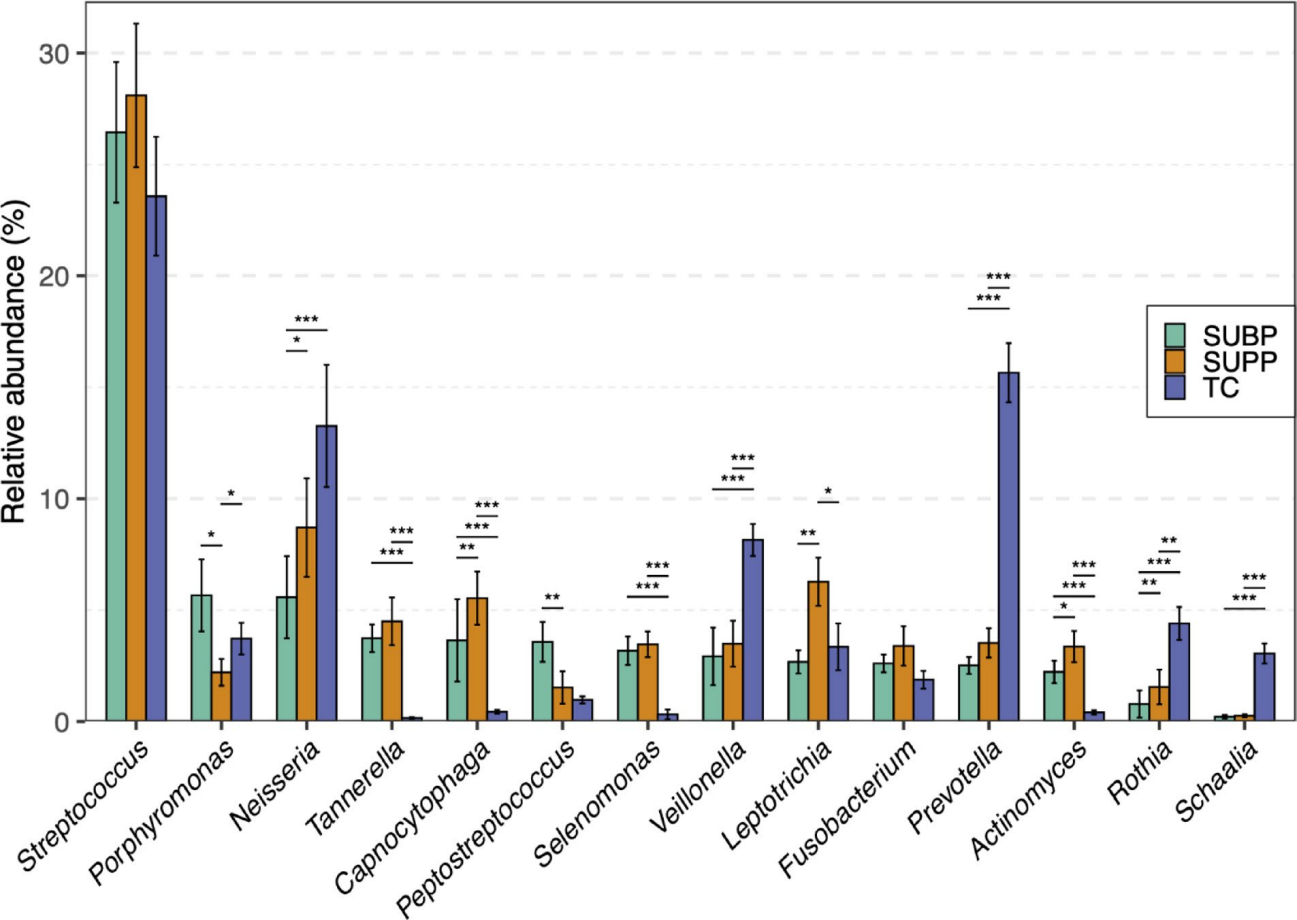
Compositional differences across subgingival plaque (SUBP), supragingival plaque (SUPP), and tongue coating (TC) microbiota. Fourteen genera with ≥ 3% mean relative abundance in any of the three microbiota are displayed. Statistical differences were identified using the Wilcoxon signed-rank test with Benjamini–Hochberg false discovery rate (FDR) adjustment. *P < 0.05, **P < 0.01, ***P < 0.001.

We compared the overall bacterial composition of SUBP samples with those of SUPP and TC samples. Both SUBP and TC samples exhibited significantly higher species diversity than SUPP samples (*P* < 0.05, Fig. 1). Principal coordinate analysis (PCoA) based on the Bray–Curtis distance at the species level demonstrated a high degree of similarity in bacterial composition between the SUBP and SUPP samples, which was significantly different from that of the TC samples (*P* < 0.01, Fig. 2, *P* < 0.001, Supplementary Fig. S1). Eight classes and 14 genera were identified as the predominant classes and genera with ≥ 3% mean relative abundance (Fig. 3 and Supplementary Fig. S2). *Porphyromonas* and *Peptostreptococcus* were significantly more abundant in the SUBP microbiota than in the SUPP microbiota (*P* < 0.05). In contrast, *Capnocytophaga*, *Leptotrichia*, and *Actinomyces* were the most abundant genera in SUPP microbiota. *Veillonella*, *Prevotella*, *Rothia*, and *Schaalia* were the predominant genera in TC microbiota. According to the species-level heatmap, *Streptococcus salivarius*, *Prevotella melaninogenica*, *Neisseria perflava*, and *Rothia mucilaginosa* were abundant and specific to the TC microbiota (Supplementary Fig. S3). Although the SUPP and SUBP microbiota share several bacterial species, a clustering approach identified a SUBP-specific group, including *Porphyromonas gingivalis*, *Streptococcus intermedius*, and *Parvimonas micra*.

In addition to compositional differences, the extent of the microbial link between the tongue and plaque microbiota was further examined. Among 206.8 ± 124.0 (mean ± SD), 94.2 ± 53.5, and 104.9 ± 59.7 ASVs observed in TC, SUPP, and SUBP, respectively, 20.1 ± 16.4, 22.4 ± 17.8, and 41.4 ± 30.3 ASVs were shared between TC and SUPP, TC and SUBP, and SUPP and SUBP, respectively, in each participant (Supplementary Fig. S4). In the TC microbiota, ASVs shared with SUPP and SUBP accounted for 13.2 ± 16.8% and 12.6 ± 14.0% (mean ± SD, Supplementary Fig. S4). Meanwhile, ASVs shared with TC occupied 37.2 ± 25.6% of SUPP microbiota and 39.6 ± 26.0% of SUBP microbiota. Between dental plaques, 62.8 ± 24.0% of SUPP and 59.2 ± 23.6% of SUBP were shared.

### Subgingival plaque-specific bacteria identification

**Fig. 4:**
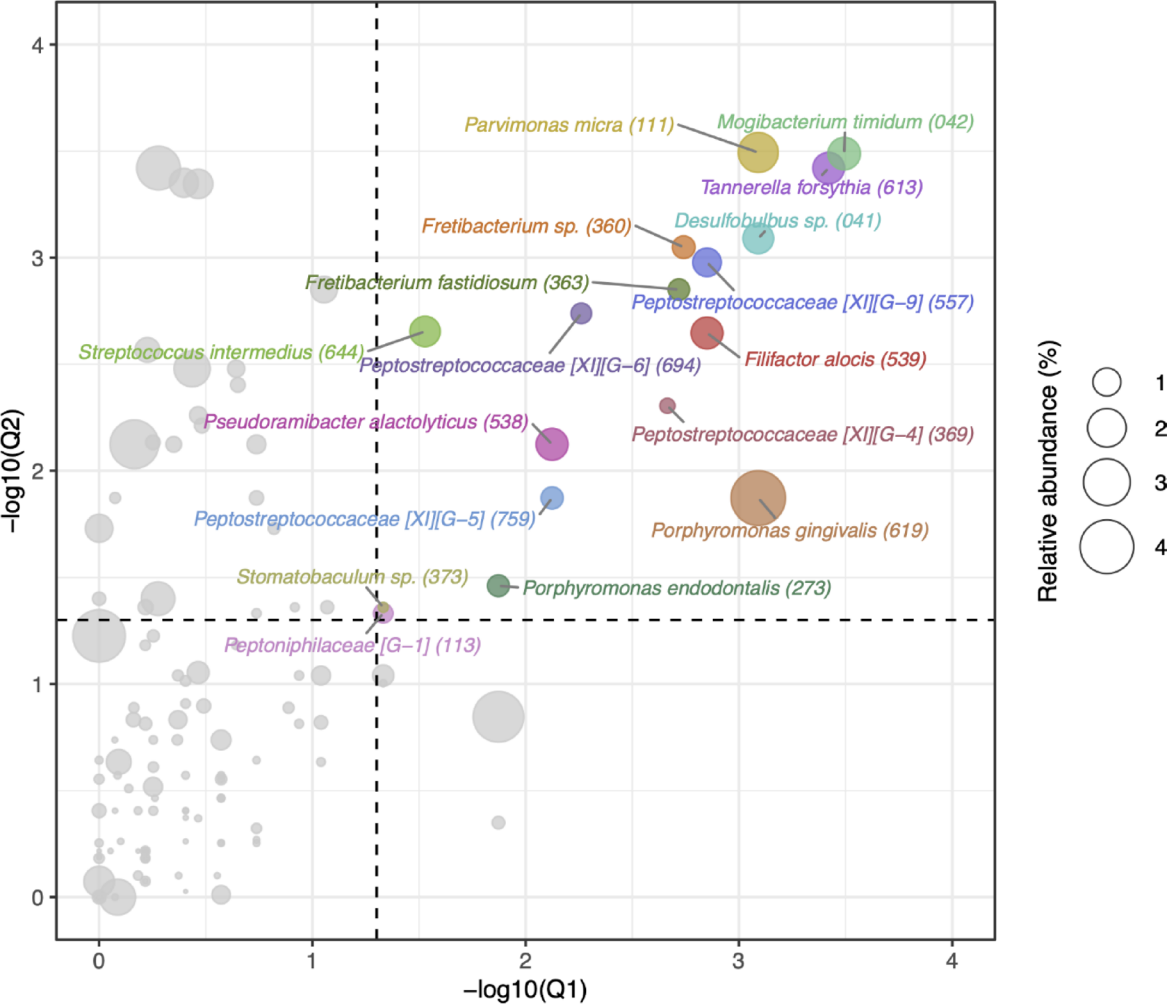
Oral niche specificity of species enriched in subgingival plaque (SUBP) microbiota. The scatter plot shows the results of Wilcoxon signed-rank tests with Benjamini–Hochberg false discovery rate (FDR) adjustment, comparing the relative abundance of bacterial species between SUBP and supragingival plaque (SUPP), as well as between SUBP and tongue coating (TC). Human microbial taxon (HMT) numbers in the eHOMD are shown in parentheses after bacterial names. The x-axis and y-axis represent the negative log10-transformed *Q*-values (adjusted *P*-values) for comparing SUBP and SUPP (Q1) and SUBP and TC (Q2), respectively. Dashed lines at − log10(0.05) indicate the significance thresholds (*Q*-value = 0.05). Colored points represent species that are significantly enriched in SUBP (*Q*-value < 0.05 for both comparisons) and have higher relative abundance in SUBP than in both SUPP and TC. Gray points represent species that have higher relative abundance in SUBP than in both SUPP and TC but do not reach statistical significance (*Q*-value ≥ 0.05 in either or both comparisons). The size of each point represents the mean relative abundance of the species in the SUBP microbiota.

**Fig. 5:**
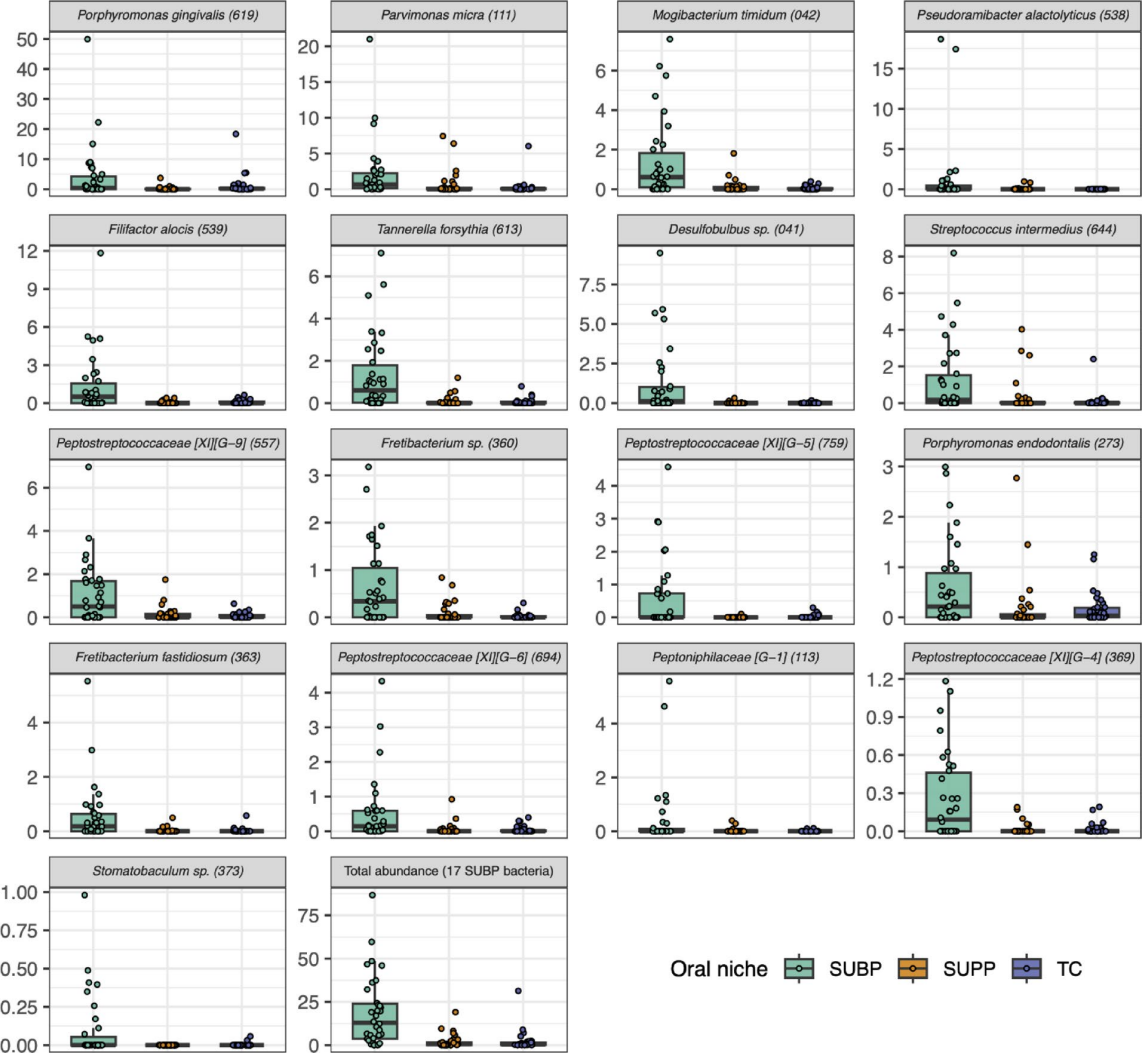
Differentially abundant species in subgingival plaque (SUBP) compared to supragingival plaque (SUPP) and tongue coating (TC). The box plots show the relative abundance (%) of 17 bacterial species that were significantly enriched in the SUBP microbiota compared to both SUPP and TC microbiota. Human microbial taxon (HMT) numbers in the eHOMD are shown in parentheses following bacterial names. Species were considered significantly enriched if they had an adjusted *P*-value < 0.05, as determined by the Wilcoxon signed-rank tests with Benjamini–Hochberg false discovery rate (FDR) adjustment.

Among the 446 species included in the species-level analysis, we focused on identifying bacteria that were specifically enriched in SUBP. Species were considered SUBP-specific if their mean relative abundance in the SUBP samples was higher than that in the SUPP and TC samples, with an FDR-adjusted *P*-value < 0.05. Thus, 17 species, including *Porphyromonas gingivalis* and *Tannerella forsythia*, met the criteria and they collectively accounted for 18.9 ± 20.2% (mean ± SD) of the SUBP microbiota (Figs. 4 and 5). Although these species were also detected in SUPP and TC samples, their relative abundance was considerably lower, at 2.1 ± 3.9% and 2.1 ± 5.6%, respectively (Fig. 5). Among the SUBP-specific species, *Mogibacterium timidum*, *T. forsythia*, *Parvimonas micra*, and *Desulfobulbus* sp. HMT-041 exhibited high SUBP specificity (*Q*-value < 0.001 for both SUBP versus SUPP and SUBP versus TC comparisons). In contrast, although *P. gingivalis* demonstrated high specificity in the comparison between SUBP and SUPP (*Q*-value < 0.001), its specificity was lower than when comparing SUBP to TC (*Q*-value < 0.05; Fig. 4).

## Discussion

We employed PacBio full-length 16S rRNA gene amplicon sequencing to accurately characterize and compare the microbial communities inhabiting the SUBP, SUPP, and TC niches in 34 participants with severe periodontitis. Our primary objective was to use the improved species-level resolution provided by this method to identify microbial species with strong specificity for the subgingival environment, which is a critical site in the pathogenesis of periodontal disease. Species-level analysis revealed a panel of 17 species that were significantly more abundant in the SUBP microbiota than in both the SUPP and TC communities (Figs. 4 and 5). These largely consisted of previously reported discriminating bacterial species in periodontitis SUBP samples^9,19^. Similar results were obtained using another comparative approach, a discriminant analysis (Supplementary Fig. S5). This group of SUBP-specific bacteria consists of obligate anaerobic bacteria, including well-known periodontopathogens such as *P. gingivalis* and *T. forsythia*^20^, and together accounted for 18.9% of the SUBP microbiota. In contrast, the relative abundance of these species was significantly lower in the SUPP (2.1%) and TC (2.1%) microbiota, underscoring their strong ecological preferences for the subgingival niche. This high-resolution profiling of SUBP microbiota provides precise information on important targets for diagnostic or therapeutic strategies for periodontal disease.

Detailed analysis of specificity profiles (Fig. 4) revealed clear differences among the 17 SUBP-specific bacteria. Four species–*Mogibacterium timidum*, *T. forsythia*, *Parvimonas micra*, and *Desulfobulbus* sp. HMT-041 exhibited particularly high SUBP specificity with low *Q*-values (< 0.001) compared to both SUPP and TC. These species are promising candidates for subgingival plaque biomarkers or may play a central role in maintaining the distinct ecology of this niche^21–25^. Interestingly, among the 17 SUBP-specific bacteria, *P. gingivalis* exhibited stronger specificity for the SUBP niche than for SUPP (*Q* < 0.001), whereas its distinction from TC was less pronounced (*Q* < 0.05), indicating a moderate degree of SUBP preference for tongue coating. In contrast, *T. forsythia* demonstrated significantly higher specificity for SUBP than for both SUPP and TC, with low *Q*-values in both comparisons. This specificity was not observed in other predominant *Tannerella* species, such as *Tannerella* species HMT-286, suggesting a more exclusive adaptation of *T. forsythia* to the subgingival environment (Supplementary Fig. S3). The ASV-level ternary plot revealed that two ASVs assigned to *T. forsythia* were specific to SUBP, whereas some of the eight ASVs assigned to *P. gingivalis* were broadly distributed toward TC (Supplementary Fig. S6). This difference in SUBP specificity suggests that, although both are key subgingival plaque species, certain *P. gingivalis* may possess a slightly broader capacity for survival under microaerophilic conditions or transient colonization in other oral niches, such as the tongue coating, compared to the more fastidious, strictly anaerobic *T. forsythia*^26–29^. These differences in niche specificity may influence how these species spread, persist, and contribute to the onset, progression, and recurrence^30^.

Our findings also suggest a microbial link across sites, in addition to site specificity. Among the three sites, ASVs composing SUPP and SUBP microbiota were highly shared (62.8 ± 24.0% of SUPP and 59.2 ± 23.6% of SUBP) (Supplementary Fig. S4). Considering that these bacteria are in contact with the tooth surface, bacterial sharing is reasonable. Meanwhile, only 13.2 ± 16.8% and 12.6 ± 14.0% of TC microbiota were shared with SUPP and SUBP microbiota, suggesting that most TC microbiota is composed of TC-specific bacteria (Supplementary Fig. S3). Interestingly, 37.2 ± 25.6% of SUPP microbiota and 39.6 ± 26.0% of SUBP microbiota were shared with TC microbiota. These results suggest a microbial link between the tongue and plaque microbiota, although they are not anatomically adjacent. We have previously demonstrated that the bacterial composition of the tongue microbiota was associated with dental plaque accumulation or dental caries experience^31–33^, suggesting that the tongue microbiota could influence the bacterial composition of the dental plaque microbiota and its pathogenicity. Considering the site-specialist hypothesis, bacteria shared with TC in the SUPP and SUBP may play an accessory role rather than being structurally integrated members of the dental plaque community^34^. The co-occurrence network analysis revealed that the major cohabiting group in SUBP samples was composed of only SUBP-specific bacteria (Supplementary Fig. S7). The functional and structural associations between tongue bacteria and dental plaque communities should be further examined in future studies. Furthermore, considering that the role of bacteria is different in each niche, it also highlights the importance of considering and modeling the distinct clinical environments of oral niches in mechanistic and antimicrobial studies.

The present study had several limitations. First, this comparative analysis was confined to plaque and tongue samples. A more comprehensive assessment and understanding of the specificity of the SUBP microbiota can be achieved by including other distinct oral niches, such as the buccal mucosa, keratinized gingiva, and hard palate, as characterized previously^35,36^. Second, dental plaque samples were pooled from multiple sites per participant. Although this approach provides an overview of the plaque microbiota, it may obscure potential site-specific differences in bacterial composition between deep and shallow periodontal or peri-implant pockets^37–40^. Future studies employing site- and depth-specific sampling strategies may reveal finer spatial heterogeneity within the subgingival microbial communities. Third, because most participants were recruited at the first visit, no standardized instructions were available before sampling, such as refraining from eating or drinking. Fourth, we could not clinically characterize the participants according to the current classification system of periodontitis^41^ owing to the lack of information on clinical attachment level, radiographic data, smoking habits, and diabetic status. Fifth, the recruitment of 34 patients with severe periodontitis from dental clinics may have introduced selection bias, potentially limiting the generalizability of our findings to broader populations or individuals with milder forms of periodontal disease. Future studies should, therefore, involve larger and more diverse cohorts to validate these observations. Sixth, because the present findings were based on the 16S rRNA gene amplicon analysis, the functional features of SUBP-specific species were not determined. Metagenomic and metatranscriptomic approaches can reveal the details of their involvement in periodontal diseases.

In conclusion, we conducted a full-length 16S rRNA gene amplicon analysis to compare bacterial communities in SUBP, SUPP, and TC at a high resolution. We identified 17 species that were significantly enriched in the subgingival niche, underscoring the selective ecological pressures within periodontal pockets. Furthermore, quantification of SUBP specificity revealed that four species, including *T. forsythia* and *P. micra*, exhibited exceptionally high SUBP specificity. Moreover, we uncovered subtle but potentially significant differences in SUBP specificity among established periodontopathogens, such as *P. gingivalis* and *T. forsythia*. These findings enhance our understanding of the subgingival microbiota in periodontitis and highlight highly specific subgingival microbial targets, which may inform the development of effective diagnostic tools or targeted therapeutic strategies for the management of periodontal disease.

## Methods

### Study participants

This study included 42 patients who visited eight dental clinics in Japan. We only included patients who had ≥ 20% of probing sites with PD ≥ 4 mm to focus on the subgingival plaque microbiota and not used antibiotics within one month before sample collection^42,43^. Among these, 13 were included in a previous study^44^. Patients who used antibiotics within 1 month before sample collection were not recruited. After excluding eight participants with missing data (*n* = 5), insufficient samples for analysis (*n* = 2), and failure to obtain appropriate PCR amplicons for sequencing (*n* = 1), a total of 34 participants (14 men and 20 women) were included in the final analysis.

### Sample collection and clinical information

Sample collection and dental examinations were performed according to a previously described protocol^45^. Plaque samples were collected from the upper half of the jaw to simplify the sampling steps. SUPP samples were collected from all tooth surfaces on the upper half of the jaw, specifically from the side with the highest number of teeth, using sterile curettes and pooled into sterile tubes. SUBP samples were subsequently obtained and pooled from the gingival crevices in the same region using sterile curettes. TC samples were collected by scraping the dorsal surface of the tongue using a sterile plastic spatula. The collected TC samples were immediately transferred into sterile tubes containing 500 µL of lysis buffer (9 mM Tris-HCl, 0.9 mM EDTA, and 1% sodium dodecyl sulfate). All samples were transported to the laboratory on ice and stored at − 80 °C until further analysis. Following sample collection, the PD and BOP were measured at six sites per tooth (mesiobuccal, midbuccal, distobuccal, mesiolingual, midlingual, and distolingual) using a periodontal probe.

### Full-length 16S rRNA gene amplicon sequencing

Different pretreatment procedures were performed for each type of sample before DNA extraction. The SUPP and SUBP samples were resuspended in 200 µL of lysis buffer. For the TC samples, 200 µL of the resuspended supernatant was transferred into new sterile tubes after the spatulas were removed following centrifugation (15,000 rpm, 15 min, 4℃). After the pretreatment procedures, the DNA was extracted from each sample using the bead-beating method described previously^46^. The full-length 16S rRNA gene, containing all variable regions, was amplified using the primers 8 F (5’-AGA GTT TGA TYM TGG CTC AG-3’) with the sample-specific 8-base barcode sequence and modified 1492R (5’-GGH TAC CTT GTT ACG ACT T-3’)^47^. PCR amplification and purification were performed as previously described^46^. The purified amplicons were sequenced on a PacBio Sequel IIe system (Pacific Biosciences; Menlo Park, CA, USA) using the Sequel II Sequencing Kit 2.0 at Kazusa Genome Technologies, and circular consensus sequencing (CCS) reads were generated. Only high-fidelity (HiFi) CCS reads with ≥ 3 full-pass subreads and ≥ 20 quality values were finally used for analysis.

### Data analysis and taxonomy assignment

The HiFi reads were primarily quality-checked using the R software (version 3.6.2; R Foundation for Statistical Computing, Vienna, Austria) and were excluded from the analysis when they exhibited < 1,000 bases or did not include the correct forward and reverse primer sequences. The remaining HiFi reads were demultiplexed by examining 8-base tag sequences at the forward end, and the forward and reverse primer sequences were trimmed. Quality filtering, denoising, and chimera filtering procedures were performed using the DADA2 pipeline (version 1.26.0) with default settings for PacBio reads, and an ASV table was subsequently created^18^. The class- and genus-level taxonomies of each sequence variant were determined using the RDP classifier with a minimum support threshold of 80% and RDP taxonomic nomenclature^48^. Sequence variants were taxonomically assigned at the species level using BLAST^49^ against 1,015 oral bacterial 16S rRNA gene sequences in eHOMD (eHOMD 16S rRNA RefSeq version 15.22)^50^. The nearest-neighbor species with ≥ 98.5% identity was selected as candidates for each sequence, and a species-level taxonomic table was generated by aggregating ASVs that were assigned to identical reference sequences in the eHOMD. ASVs without hits were excluded from the species-level analysis.

### Ethics statement

The Ethics Committee of Kyushu University approved the present study and the procedure for obtaining informed consent (approval number: 2019 -105). All experiments in this study were performed in accordance with the relevant guidelines and regulations, including the principles of the Declaration of Helsinki. Written informed consent was obtained from all the participants prior to inclusion in the study.

### Statistical analysis

All statistical analyses were performed using the R software. For all multiple comparisons, *P*-values were adjusted using the Benjamini–Hochberg method to control the false discovery rate (FDR). An FDR-adjusted *P*-value (*Q*-value) < 0.05 was considered statistically significant. Alpha diversity was assessed using the number of observed species calculated following rarefaction of 1,000 reads per sample using the diversity function in the vegan package. Differences in the number of species in the three oral niches (SUBP, SUPP, and TC) were compared using the Wilcoxon signed-rank test. Beta diversity was evaluated using Bray–Curtis distances based on a log-transformed species-level table. Significant differences in the overall microbiota structure among the niches were assessed by performing a permutational multivariate analysis of variance using the adonis2 function in the vegan package with 999 permutations. Community similarities were compared by comparing the inter-niche log-transformed Bray–Curtis distances using the Wilcoxon signed-rank test. In this study, ASVs detected in the TC and SUPP microbiota were defined as TC-SUPP-shared ASVs for each participant, and ASVs shared between the TC and SUBP microbiota were regarded as TC-SUBP-shared ASVs. The number and total abundance of the shared ASVs were calculated for each participant. Differential abundance analysis was performed at both the genus and species levels. Pairwise comparisons were performed using the Wilcoxon signed-rank test to identify genera with significantly different abundances among the three oral niches. SUBP-specific species were identified by comparing the relative abundance of each species in SUBP samples with its abundance in SUPP and TC samples using Wilcoxon signed-rank tests.

## Supplementary Information

Below is the link to the electronic supplementary material.

Supplementary Material 1

## Data Availability

The sequence data were deposited in the DDBJ BioProject database under accession number PRJDB35957, accessible at https://www.ncbi.nlm.nih.gov/bioproject/?term=PRJDB35957.

## References

[1] Peres, M. A. et al. Oral diseases: A global public health challenge. *Lancet* **394**, 249–260 (2019). 31327369 10.1016/S0140-6736(19)31146-8

[2] Hernández, M. et al. Host-pathogen interactions in progressive chronic periodontitis. *J. Dent. Res.* **90**, 1164–1170 (2011). 21471325 10.1177/0022034511401405

[3] Hajishengallis, G. Periodontitis: From microbial immune subversion to systemic inflammation. *Nat. Rev. Immunol.* **15**, 30–44 (2015). 25534621 10.1038/nri3785 PMC4276050

[4] Abusleme, L. et al. The subgingival microbiome in health and periodontitis and its relationship with community biomass and inflammation. *ISME J.* **7**, 1016–1025 (2013). 23303375 10.1038/ismej.2012.174 PMC3635234

[5] Curtis, M. A., Diaz, P. I. & Van Dyke, T. E. The role of the microbiota in periodontal disease. *Periodontol. 2000* **83**, 14–25 (2020). 10.1111/prd.12296 32385883

[6] Oliveira, R. R. D. S. et al. Levels of candidate periodontal pathogens in subgingival biofilm. *J. Dent. Res.* **95**, 711–718 (2016). 26936213 10.1177/0022034516634619 PMC4924544

[7] Tsai, C. Y. et al. Subgingival microbiota in individuals with severe chronic periodontitis. *J. Microbiol. Immunol. Infect.* **51**, 226–234 (2018). 27262209 10.1016/j.jmii.2016.04.007

[8] Van Dyke, T. E., Bartold, P. M. & Reynolds, E. C. The nexus between periodontal inflammation and dysbiosis. *Front. Immunol.* **11**, 511 (2020). 32296429 10.3389/fimmu.2020.00511 PMC7136396

[9] Chen, T., Marsh, P. D. & Al-Hebshi, N. N. SMDI: An index for measuring subgingival microbial dysbiosis. *J. Dent. Res.* **101**, 331–338 (2022). 34428955 10.1177/00220345211035775 PMC8982011

[10] Stephen, A. S. et al. Interdental and subgingival microbiota may affect the tongue microbial ecology and oral malodour in health, gingivitis and periodontitis. *J. Periodontal Res.* **56**, 1174–1184 (2021). 34486723 10.1111/jre.12931

[11] Johnson, J. S. et al. Evaluation of 16S rRNA gene sequencing for species and strain-level microbiome analysis. *Nat. Commun.* **10**, 5029 (2019). 31695033 10.1038/s41467-019-13036-1 PMC6834636

[12] Buetas, E. et al. Full-length 16S rRNA gene sequencing by PacBio improves taxonomic resolution in human microbiome samples. *BMC Genom.* **25**, 310 (2024). 10.1186/s12864-024-10213-5 PMC10964587 38528457

[13] Diao, J. et al. Potential roles of the free salivary Microbiome dysbiosis in periodontal diseases. *Front Cell. Infect. Microbiol* **11** (2021). 10.3389/fcimb.2021.711282 PMC8493099 34631597

[14] Kageyama, S. et al. High-resolution detection of translocation of oral bacteria to the gut. *J. Dent. Res.* **102**, 752–758 (2023). 37204134 10.1177/00220345231160747 PMC10288163

[15] Buetas, E., Jordán-López, M., López-Roldán, A., Mira, A. & Carda-Diéguez, M. Impact of periodontitis on the leakage of oral bacteria to the gut. *J. Dent. Res.* **103**, 289–297 (2024). 38193290 10.1177/00220345231221709

[16] Yu, P. S. et al. Microbiome of periodontitis and peri-implantitis before and after therapy: Long-read 16S rRNA gene amplicon sequencing. *J. Periodontal Res.* **59**, 657–668 (2024). 38718089 10.1111/jre.13269

[17] Dieckow, S. et al. Structure and composition of early biofilms formed on dental implants are complex, diverse, subject-specific and dynamic. *NPJ Biofilms Microbiomes* **10**, 155 (2024). 39719447 10.1038/s41522-024-00624-3 PMC11668855

[18] Callahan, B. J. et al. DADA2: High-resolution sample inference from illumina amplicon data. *Nat. Methods* **13**, 581–583 (2016). 27214047 10.1038/nmeth.3869 PMC4927377

[19] Chew, R. J. J., Tan, K. S., Chen, T., Al-Hebshi, N. N. & Goh, C. E. Quantifying periodontitis-associated oral dysbiosis in tongue and saliva microbiomes-an integrated data analysis. *J. Periodontol.* **96**, 55–66 (2025). 39007741 10.1002/JPER.24-0120 PMC11787769

[20] Socransky, S. S., Haffajee, A. D., Cugini, M. A., Smith, C. & Kent, R. L. Microbial complexes in subgingival plaque. *J. Clin. Periodontol.* **25**, 134–144 (1998). 9495612 10.1111/j.1600-051x.1998.tb02419.x

[21] Marchesan, J. T. et al. Association of synergistetes and cyclodipeptides with periodontitis. *J. Dent. Res.* **94**, 1425–1431 (2015). 26198391 10.1177/0022034515594779

[22] Lafaurie, G. I. et al. Differences in the subgingival microbiome according to stage of periodontitis: A comparison of two geographic regions. *PLoS One*. **17**, e0273523 (2022). 35998186 10.1371/journal.pone.0273523 PMC9398029

[23] Herrera, B. S. et al. Pursuing new periodontal pathogens with an improved RNA-oligonucleotide quantification technique (ROQT). *Arch. Oral Biol.* **152**, 105721 (2023). 37196563 10.1016/j.archoralbio.2023.105721

[24] Al-hebshi, N. N., Al-Alimi, A., Taiyeb-Ali, T. & Jaafar, N. Quantitative analysis of classical and new putative periodontal pathogens in subgingival biofilm: A case-control study. *J. Periodontal Res.* **50**, 320–329 (2015). 25040261 10.1111/jre.12210

[25] Antezack, A., Etchecopar-Etchart, D., La Scola, B. & Monnet-Corti, V. New putative periodontopathogens and periodontal health-associated species: A systematic review and meta-analysis. *J. Periodontal Res.* **58**, 893–906 (2023). 37572051 10.1111/jre.13173

[26] Ximénez-Fyvie, L. A., Haffajee, A. D. & Socransky, S. S. Microbial composition of supra- and subgingival plaque in subjects with adult periodontitis. *J. Clin. Periodontol.* **27**, 722–732 (2000). 11034118 10.1034/j.1600-051x.2000.027010722.x

[27] Mager, D. L., Ximenez-Fyvie, L. A., Haffajee, A. D. & Socransky, S. S. Distribution of selected bacterial species on intraoral surfaces. *J. Clin. Periodontol.* **30**, 644–654 (2003). 12834503 10.1034/j.1600-051x.2003.00376.x

[28] Lewis, J. P., Iyer, D. & Anaya-Bergman, C. Adaptation of Porphyromonas gingivalis to microaerophilic conditions involves increased consumption of formate and reduced utilization of lactate. *Microbiology*. **155**, 3758–3774 (2009). 19684063 10.1099/mic.0.027953-0 PMC2888126

[29] Galimanas, V. et al. Bacterial community composition of chronic periodontitis and novel oral sampling sites for detecting disease indicators. *Microbiome* **2**, 32 (2014). 25225610 10.1186/2049-2618-2-32 PMC4164120

[30] Yama, K. et al. Dysbiosis of oral microbiome persists after dental treatment-induced remission of periodontal disease and dental caries. *mSystems* **8**, e0068323 (2023). 37698410 10.1128/msystems.00683-23 PMC10654066

[31] Kageyama, S. et al. Establishment of tongue microbiota by 18 months of age and determinants of its microbial profile. *mBio* **14**, e0133723 (2023). 37819142 10.1128/mbio.01337-23 PMC10653898

[32] Zhang, D. et al. Tongue microbiota composition and dental caries experience in primary school children. *mSphere* **6**, e01252–e01220 (2021). 33910998 10.1128/mSphere.01252-20 PMC8092142

[33] Asakawa, M. et al. Tongue microbiota and oral health status in community-dwelling elderly adults. *mSphere* **3**, e00332–e00318 (2018). 30111628 10.1128/mSphere.00332-18 PMC6094060

[34] Mark Welch, J. L., Dewhirst, F. E. & Borisy, G. G. Biogeography of the oral microbiome: The site-specialist hypothesis. *Annu. Rev. Microbiol.* **73**, 335–358 (2019). 31180804 10.1146/annurev-micro-090817-062503 PMC7153577

[35] Segata, N. et al. Composition of the adult digestive tract bacterial microbiome based on seven mouth surfaces, tonsils, throat and stool samples. *Genome Biol.* **13**, R42 (2012). 22698087 10.1186/gb-2012-13-6-r42 PMC3446314

[36] Eren, A. M., Borisy, G. G., Huse, S. M. & Mark Welch, J. L. Oligotyping analysis of the human oral microbiome. *Proc. Natl. Acad. Sci. U. S. A.* **111**, E2875–E2884 (2014). 24965363 10.1073/pnas.1409644111 PMC4104879

[37] Tamashiro, R. et al. Stability of healthy subgingival microbiome across space and time. *Sci. Rep.* **11**, 23987 (2021). 34907334 10.1038/s41598-021-03479-2 PMC8671439

[38] Pérez-Chaparro, P. J. et al. Do different probing depths exhibit striking differences in microbial profiles? *J. Clin. Periodontol.* **45**, 26–37 (2018). 28871594 10.1111/jcpe.12811

[39] Kröger, A. et al. The severity of human peri-implantitis lesions correlates with the level of submucosal microbial dysbiosis. *J. Clin. Periodontol.* **45**, 1498–1509 (2018). 30341964 10.1111/jcpe.13023

[40] Joshi, A. A. et al. The submucosal microbiome correlates with peri-implantitis severity. *J. Dent. Res.* 220345251352809. 10.1177/00220345251352809 (2025). 10.1177/00220345251352809 PMC12861548 40719760

[41] Tonetti, M. S., Greenwell, H. & Kornman, K. S. Staging and grading of periodontitis: Framework and proposal of a new classification and case definition. *J. Periodontol.* **89** (Suppl 1), S159–S172 (2018). 29926952 10.1002/JPER.18-0006

[42] Oral health surveys: Basic methods—5th edition. World Health Organization (2013).

[43] JSP Clinical Practice Guideline for the Periodontal Treatment 2015. *The Japanese Society of Periodontology * (2015).

[44] Ma, J. et al. Clinical utility of subgingival plaque-specific bacteria in salivary microbiota for detecting periodontitis. *PLoS One*. **16**, e0253502 (2021). 34170942 10.1371/journal.pone.0253502 PMC8232462

[45] Kageyama, S. et al. Relative abundance of total subgingival plaque-specific bacteria in salivary microbiota reflects the overall periodontal condition in patients with periodontitis. *PLoS One*. **12**, e0174782 (2017). 28369125 10.1371/journal.pone.0174782 PMC5378373

[46] Kageyama, S. et al. High-level acquisition of maternal oral bacteria in formula-fed infant oral microbiota. *mBio* **13**, e0345221 (2022). 35038919 10.1128/mbio.03452-21 PMC8764541

[47] Weisburg, W. G., Barns, S. M., Pelletier, D. A. & Lane, D. J. 16S ribosomal DNA amplification for phylogenetic study. *J. Bacteriol.* **173**, 697–703 (1991). 1987160 10.1128/jb.173.2.697-703.1991 PMC207061

[48] Wang, Q., Garrity, G. M., Tiedje, J. M. & Cole, J. R. Naïve Bayesian classifier for rapid assignment of rRNA sequences into the new bacterial taxonomy. *Appl. Environ. Microbiol.* **73**, 5261–5267 (2007). 17586664 10.1128/AEM.00062-07 PMC1950982

[49] Altschul, S. F., Gish, W., Miller, W., Myers, E. W. & Lipman, D. J. Basic local alignment search tool. *J. Mol. Biol.* **215**, 403–410 (1990). 2231712 10.1016/S0022-2836(05)80360-2

[50] Chen, T. et al. The human oral microbiome database: A web accessible resource for investigating oral microbe taxonomic and genomic information. *Database J. Biol. Databases Curation* **2010**, baq013 (2010). 10.1093/database/baq013 PMC2911848 20624719

