# Supplementary material for: Characterization of subgingival plaque microbiota in patients with severe periodontitis using full-length 16S rRNA gene sequencing
Source: Sci Rep. 2025 Dec 2;16:450. doi: 10.1038/s41598-025-30064-8 (PMC12775462; doi:10.1038/s41598-025-30064-8)
Supplement: Supplementary file 1 — Supplementary Material 1 [file 41598_2025_30064_MOESM1_ESM.pdf]

Characterization of subgingival plaque microbiota in patients with severe periodontitis using full-length 16S rRNA gene sequencing

Jiale Ma[1], Shinya Kageyama[1]*, Mikari Asakawa[1], Michiko Furuta[1], Yoshihisa Yamashita[1], Toru Takeshita[1]

[1]Section of Preventive and Public Health Dentistry, Division of Oral Health, Growth and Development, Faculty of Dental Science, Kyushu University, Fukuoka, Japan.

*Corresponding author

E-mail: s.kageyama@dent.kyushu-u.ac.jp (S.K.)

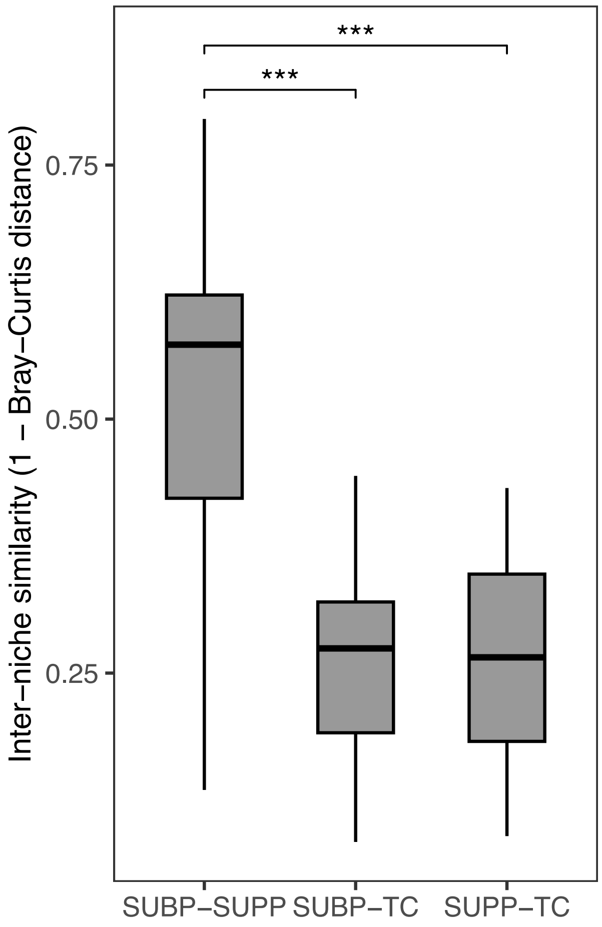

Supplementary Figure S1. Compositional similarity across oral niches. The bacterial compositional similarity across different oral niches (inter-niche) was compared among subgingival plaque (SUBP), supragingival plaque (SUPP), and tongue coating (TC). Statistical significance was assessed using the Wilcoxon signed-rank test with Benjamini–Hochberg false discovery rate (FDR) adjustment. ***$P < 0.001$.

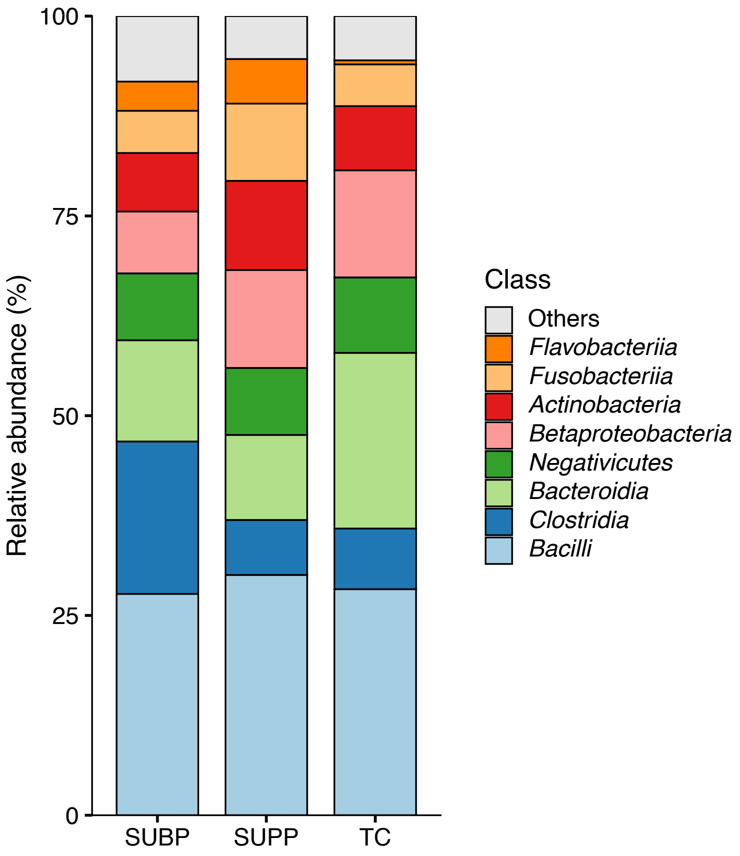

Supplementary Figure S2. Compositional differences across oral niches. Eight classes with ≥ 3% mean relative abundance in any of the three microbiota are displayed.

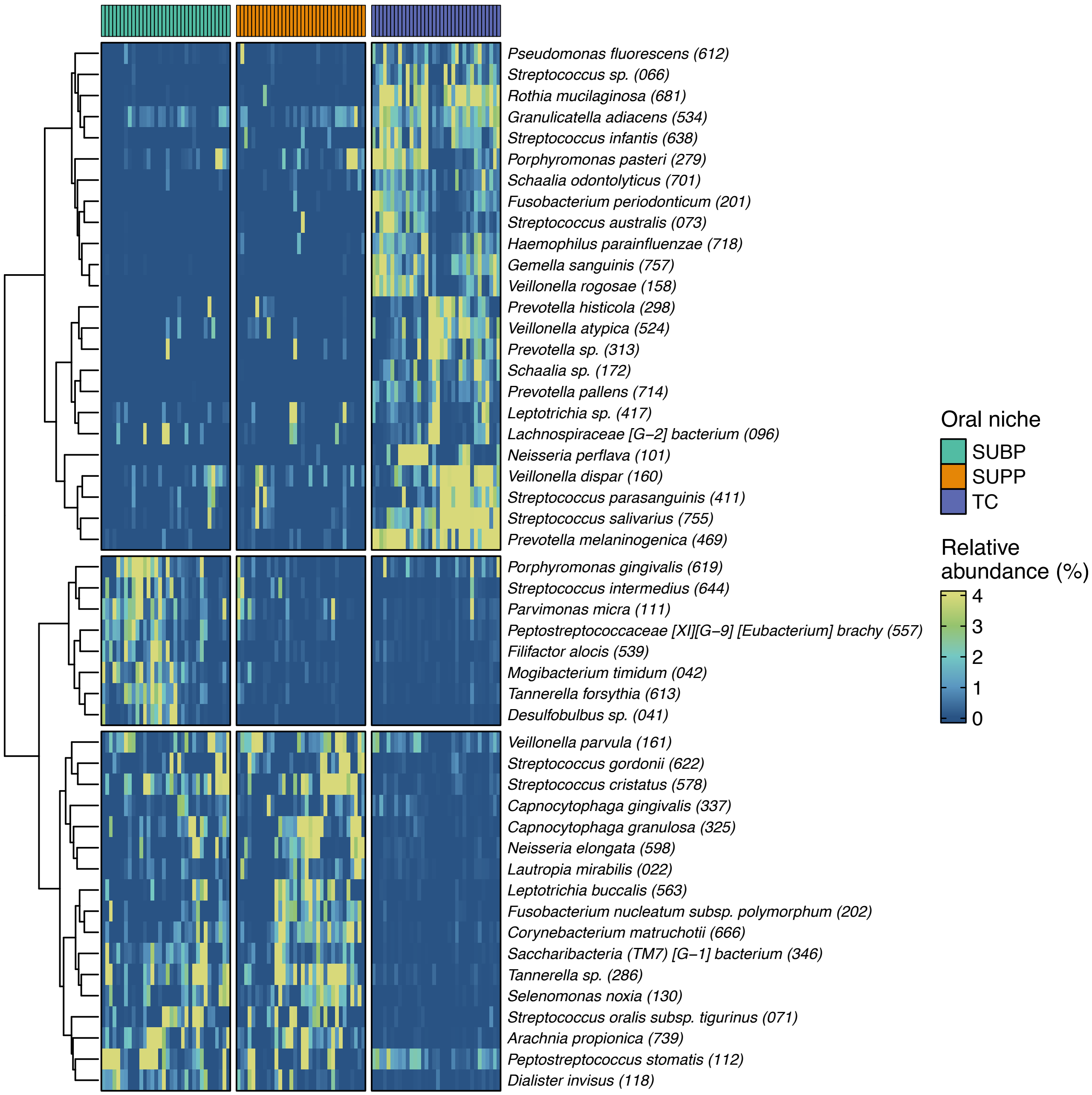

Supplementary Figure S3. Heatmap of bacterial composition across oral niches. Forty-nine species with a mean relative abundance ≥ 1% and a detection rate ≥ 50% in any of the three microbiota are shown. Human microbial taxon (HMT) numbers in the eHOMD are depicted in parentheses after bacterial names. The relative abundance of each species is represented by color intensity. The hierarchical clustering of species is performed based on the Bray–Curtis distance.

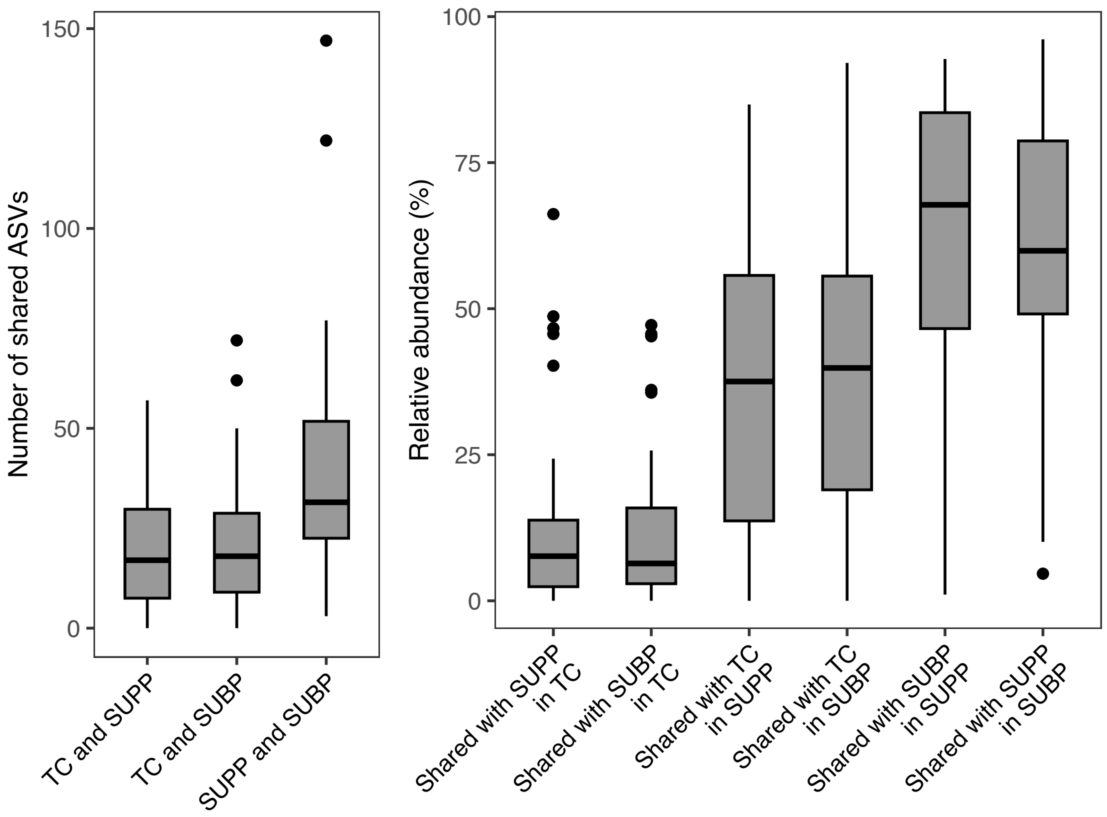

Supplementary Figure S4. Microbial link across oral niches. The number of shared ASVs and the total relative abundance of shared ASVs are shown.

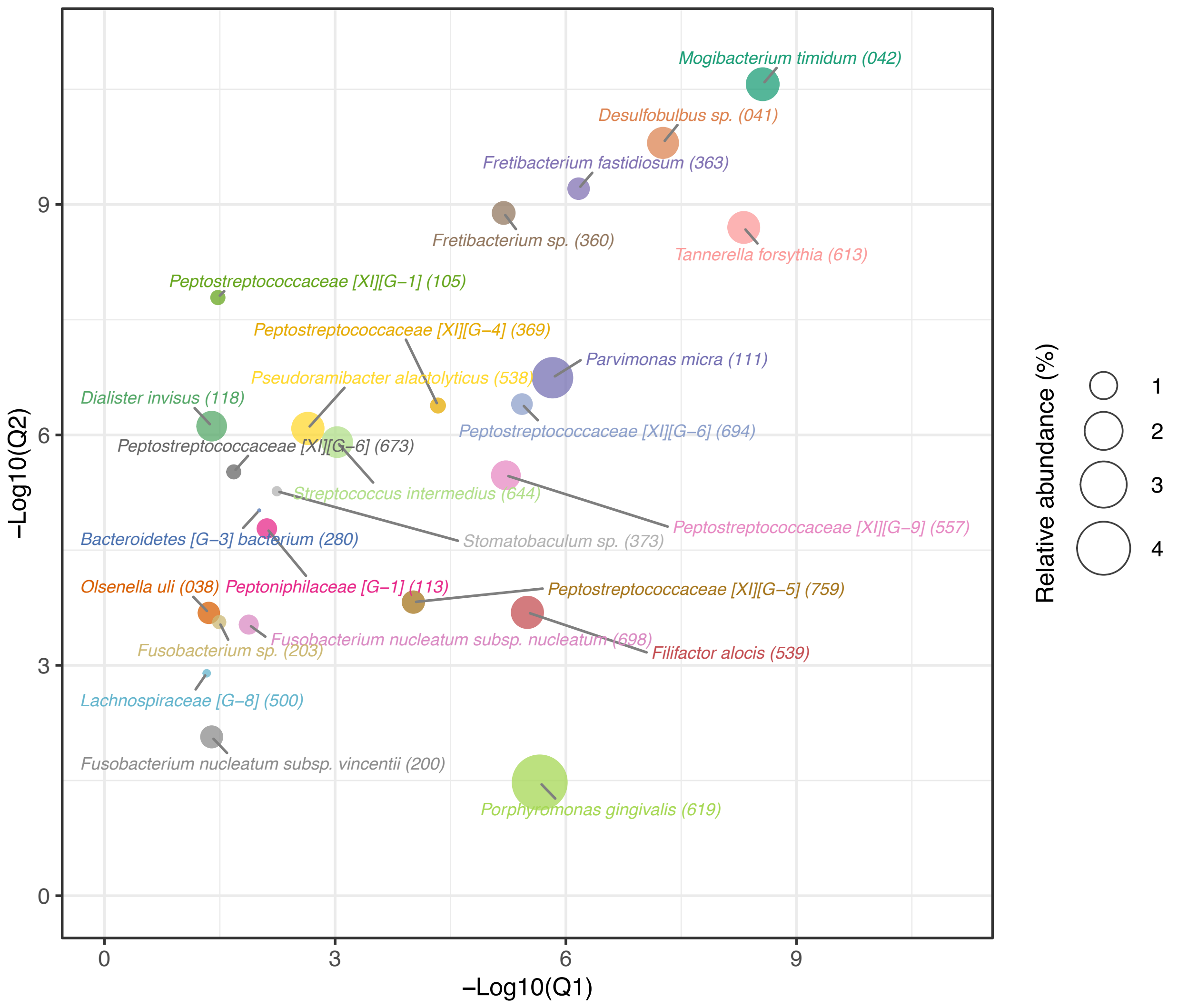

Supplementary Figure S5. Oral niche specificity of species enriched in subgingival plaque (SUBP) microbiota. The scatter plot shows the results of the MaAsLin2 analysis with a *Q*-value (adjusted *P*-value) significance threshold < 0.05. The relative abundances of bacterial species between SUBP and supragingival plaque (SUPP), as well as between SUBP and tongue coating (TC), are compared. Human microbial taxon (HMT) numbers in the eHOMD are shown in parentheses after bacterial names. The x-axis and y-axis represent the negative log10-transformed *Q*-values for comparing SUBP and SUPP (Q1) and SUBP and TC (Q2), respectively. Colored points represent species that are significantly enriched in SUBP (*Q*-value < 0.05 for both comparisons) and have higher relative abundance in SUBP than in both SUPP and TC. The size of each point represents the mean relative abundance of the species in the SUBP microbiota.

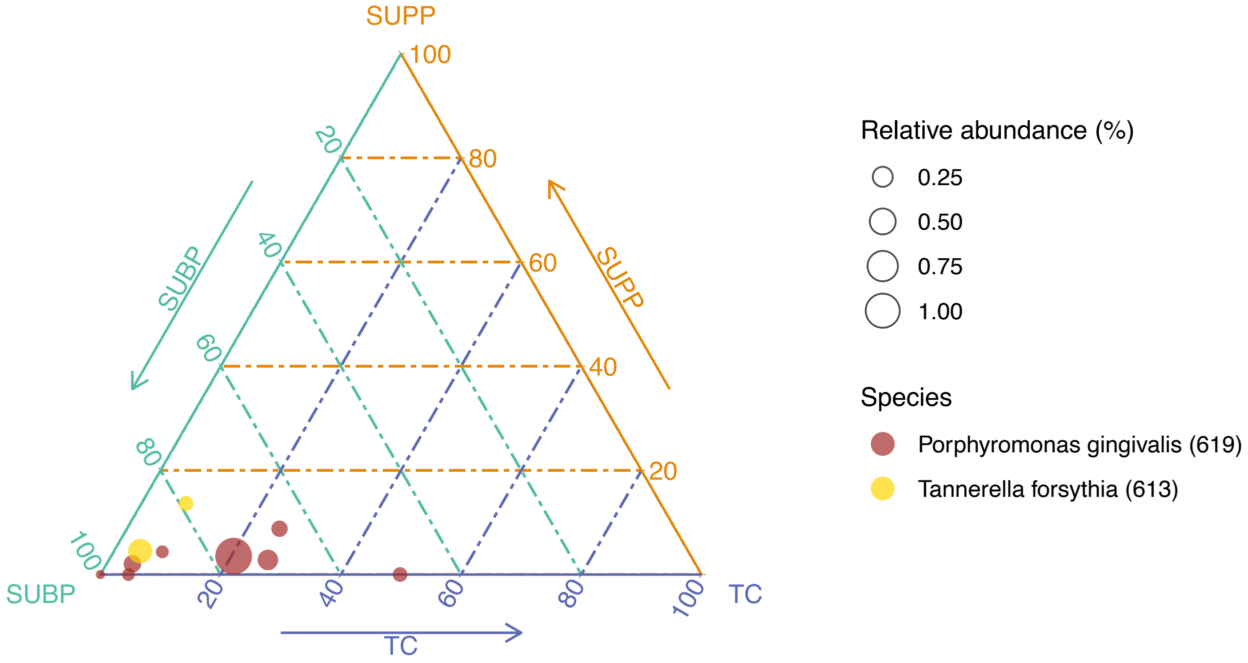

Supplementary Figure S6. Ternary plot showing site-specificity of *Porphyromonas gingivalis* and *Tannerella forsythia* at the ASV-level. Ten ASVs with a mean relative abundance ≥ 0.1% in any of the three oral microbiota are shown. Human microbial taxon (HMT) numbers in the eHOMD are shown in parentheses after bacterial names. The size of each point represents the mean relative abundance across oral niches.

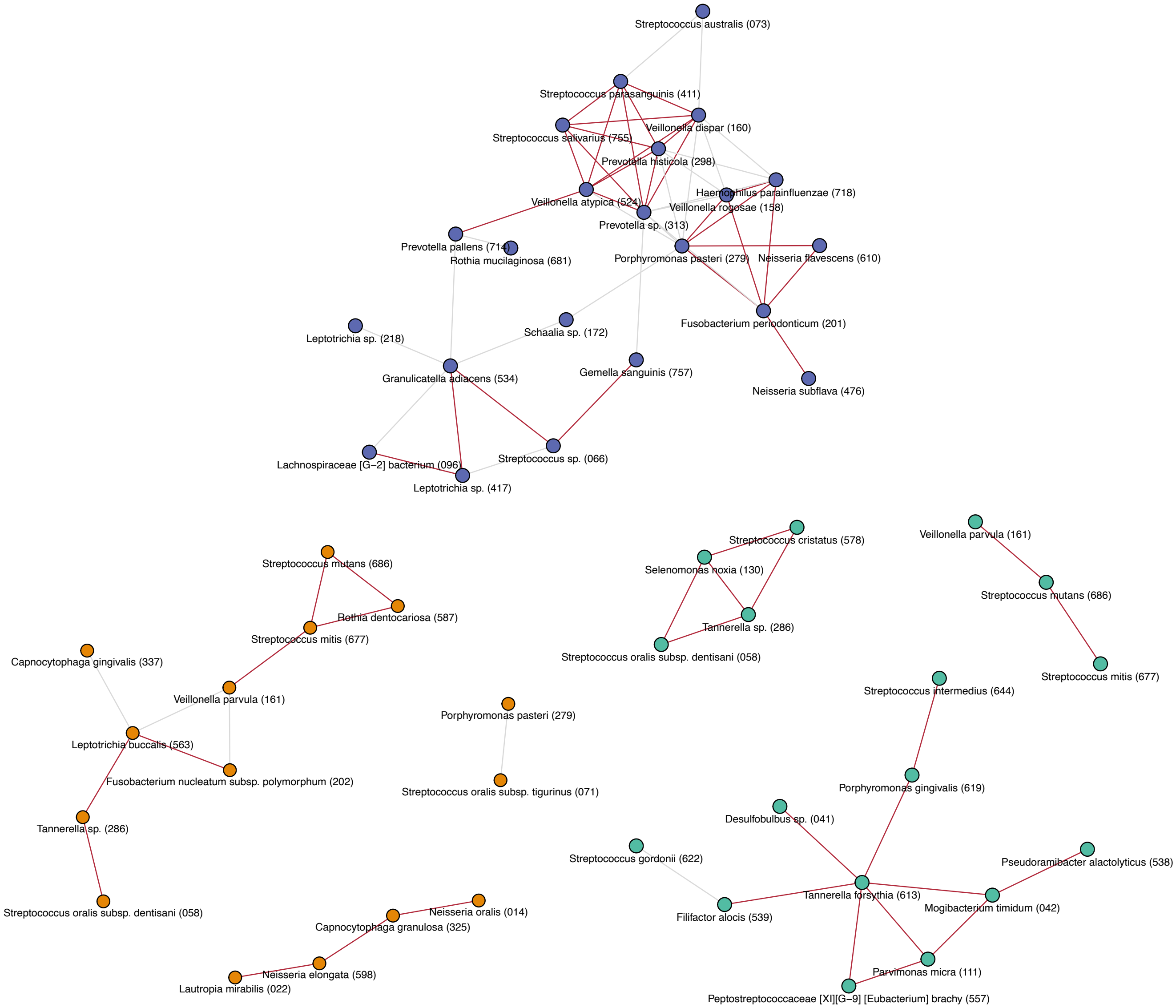

Supplementary Figure S7. Co-occurrence networks built from SparCC correlation coefficients in each oral niche at the species-level. Co-occurrence network analyses are performed with a mean relative abundance ≥ 1% and a detection rate ≥ 20% in each of the three microbiota. Human microbial taxon (HMT) numbers in the eHOMD are shown in parentheses after bacterial names. Each node corresponds to a distinct species, and correlations with absolute values greater than 0.4 and *P*-values less than 0.01 are represented as edges. Positive correlations are shown in red, whereas negative correlations are shown in gray. Nodes colored in purple, orange, and green correspond to the tongue coating (TC), supragingival plaque (SUPP), and subgingival plaque (SUBP) microbiotas, respectively.
